# Exploring Copper Oxide and Copper Sulfide for Non-Enzymatic Glucose Sensors: Current Progress and Future Directions

**DOI:** 10.3390/mi14101849

**Published:** 2023-09-27

**Authors:** Nonkululeko Miya, Lerato F. Eugeni Machogo-Phao, Bulelwa Ntsendwana

**Affiliations:** DSI/Mintek Nanotechnology Innovation Centre, Advanced Materials Division, Mintek, Private Bag X3015, Randburg 2125, South Africa; nonkululekom@mintek.co.za (N.M.); bulelwan@mintek.co.za (B.N.)

**Keywords:** copper oxide, copper sulfide, non-enzymatic, glucose, electrochemical, sensor

## Abstract

Millions of people worldwide are affected by diabetes, a chronic disease that continuously grows due to abnormal glucose concentration levels present in the blood. Monitoring blood glucose concentrations is therefore an essential diabetes indicator to aid in the management of the disease. Enzymatic electrochemical glucose sensors presently account for the bulk of glucose sensors on the market. However, their disadvantages are that they are expensive and dependent on environmental conditions, hence affecting their performance and sensitivity. To meet the increasing demand, non-enzymatic glucose sensors based on chemically modified electrodes for the direct electrocatalytic oxidation of glucose are a good alternative to the costly enzymatic-based sensors currently on the market, and the research thereof continues to grow. Nanotechnology-based biosensors have been explored for their electronic and mechanical properties, resulting in enhanced biological signaling through the direct oxidation of glucose. Copper oxide and copper sulfide exhibit attractive attributes for sensor applications, due to their non-toxic nature, abundance, and unique properties. Thus, in this review, copper oxide and copper sulfide-based materials are evaluated based on their chemical structure, morphology, and fast electron mobility as suitable electrode materials for non-enzymatic glucose sensors. The review highlights the present challenges of non-enzymatic glucose sensors that have limited their deployment into the market.

## 1. Introduction

**Figure 1:**
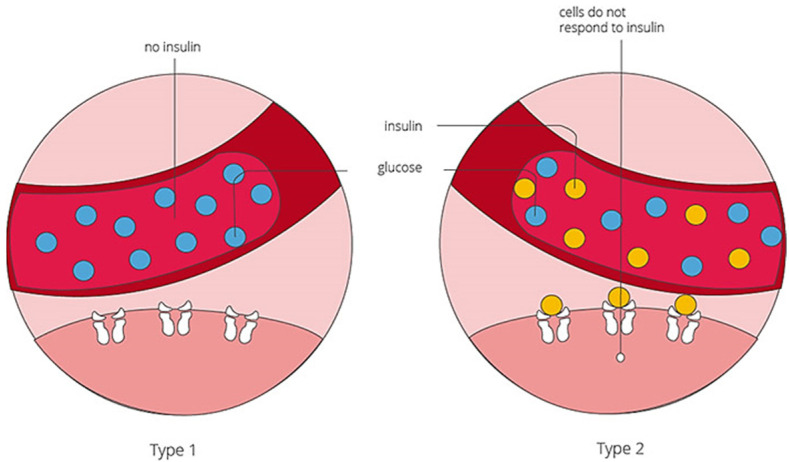
Diabetes mellitus types [6].

Diabetes is the fastest-growing disease globally, and the number of people living with diabetes continues to increase every year worldwide. Type 1 diabetes is caused by low or no insulin production by the pancreas and Type 2 diabetes results from the body’s inability to produce enough insulin or its ineffective use of insulin (Figure 1). Type 2 diabetes is the most prevalent of the two, affecting approximately 95% of people with diabetes [1]. According to the WHO, an estimated 422 million people worldwide have diabetes, with the majority living in low- and middle-income countries, and 1.5 million deaths directly related to diabetes each year [2]. As such, there is a growing need for reliable, cost-effective, easy-to-use rapid glucose biosensors, especially in developing countries, to assist the public health sector and those with limited resources to monitor glucose levels. There has been an increase in biosensor research, specifically that of electrochemical glucose sensors, and it is becoming a fast-growing field. Enzyme glucose oxidase (GOx)-based biosensors are on-the-market enzymatic sensors that rely on either amperometric measurement of consumed oxygen/produced H_2_O_2_ (first-generation sensors) or toxic mediators (second-generation sensors) [3,4]. However, studies have also shown that various conditions such as temperature, humidity, and pH affect the sensing performance of enzymatic sensors, hence the gradual movement from enzyme-based biosensors (part of the third generation of sensors) to reliable materials in non-enzymatic detectors [5].

The presence of electrochemical interferents (also electro-active) in blood samples, such as salicylic acid, dopamine, ibuprofen, and ascorbic acid [7], has in the past caused a false high reading of glucose by donating electrons not derived from glucose, which generates a high current response [8]. To date, electrochemical sensors for glucose are the most commercialized for diabetes maintenance [5]. Electrochemical glucose biosensors are based on catalytic glucose oxidation through an enzyme or nanomaterial, generating an electron flow that is measured through an electric signal. The sensing performance of selective and highly responsive non-enzymatic glucose sensors is highly reliant on the materials used for electrode fabrication and/or modification [9]. The advantage of developing non-enzymatic sensors over enzymatic sensors is that they have been reported to be functional for more than 30 days in undiluted whole blood after sterilization, showing enhanced stability and selectivity [10]. This has opened new avenues to evaluate various nanomaterials that have desirable sensitivity, reliability, and economic value. Copper-based nanomaterials such as copper oxides and copper sulfides have been studied due to their outstanding properties, abundance, and catalytic ability as electrode materials and modifiers for the non-enzymatic detection of glucose. This review highlights and summarizes the progress made in copper oxide and copper sulfide for non-enzymatic glucose sensors. It also evaluates their structural, morphological, and catalytic properties relative to their performance towards glucose detection. We also discuss the limitations and the future outlook of these nanomaterials for application in glucose sensing.

## 2. Enzymatic to Non-Enzymatic Glucose Sensors

The introduction of an enzymatic amperometric glucose sensor by Clark, operated by the immobilization of GOx on a Pt electrode, was the first generation of electrochemical glucose sensors [11]. Enzymatic sensors recognize and react with the target substance using biological molecules, usually enzymes. The sensor’s enzyme recognizes and attaches to the target compound (substrate). This interaction typically results in a chemical change in the enzyme. The change can be measured and converted into an electrical, optical, or other quantifiable signal via the transducer. The glucose sensor was based on monitoring oxygen consumption, which is proportional to the cell current. The increase in current (oxygen concentration) is proportional to the glucose concentration. The electrochemical reduction occurred at the Pt electrode. The other methods involved measuring the gluconic acid produced with a pH meter or measuring the H_2_O_2_ production with a peroxide sensor. The first-generation sensors have the following limitations: background oxygen interference during the reaction; restricted oxygen solubility in biological fluids, which limits enzymatic reactions; and a high operating potential required for monitoring H_2_O_2,_ causing electroactive interference at that potential [12]. To improve the first-generation glucose sensor, oxygen as a mediator had to be replaced. This was done in the second generation of glucose sensors using an artificial electron acceptor/mediator doped into the enzyme membrane, facilitating the flow of electrons between the redox center of the enzyme and the electrode surface. These mediators can also form covalent bonds with the amine groups present in the structure of GOx, through crosslinking, with thiol and aldehyde functional groups, leading to enhanced electrode stability. 

**Figure 2:**
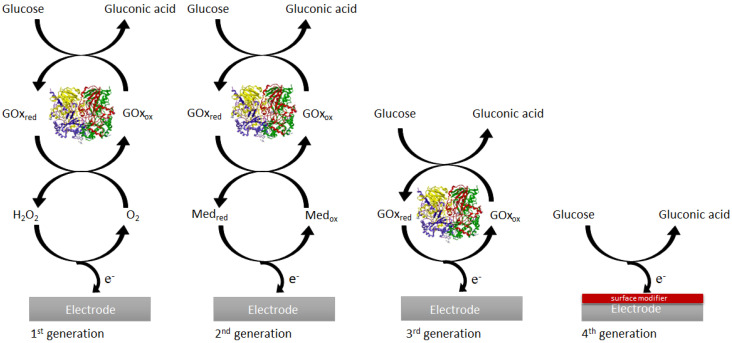
Generations of glucose sensors from enzymatic to non-enzymatic.

The glucose sensor relied on the mediator to transport electrons to and from the enzyme’s active site [13]. The limitation of this generation of glucose sensors is that the immobilized mediators suffer from a limited range of motion. The third generation of glucose sensors is based on the use of an electrode for direct electrical communication with the enzyme without mediators. The sensor relies on direct electron transfer, which depends on the enzyme’s redox center and electrode surface distance. The drawback of the third-generation glucose sensor is its dependence on the enzyme’s activity. Another drawback to using enzymes is the dependence on the enzymatic layer thickness, which results in signal dampening or loss [14]. The development of glucose sensors, which eliminate the use of enzymes for the transfer of electrons directly from the enzyme to the electrode (first generation), was successful through the third generation of enzymatic glucose sensors, where the enzyme was immobilized onto the electrode [5]. The recent development of non-enzymatic glucose sensors (fourth generation) has been the incorporation of nanomaterials to enhance the rate of electron transfer (Figure 2). Non-enzymatic sensors do not rely on biological molecules for detection. Instead, they make use of the unique physicochemical features of the target substance. Non-enzymatic sensors are frequently based on the electrochemical characteristics of the target compound. They can use metal oxides, nanoparticles, or other conductive materials. The target compound reacts directly with the sensor material, creating a change in conductivity or electrochemical potential. This change is then measured by the transducer. Considering the limitations of enzymatic glucose sensors from the first to the third generation, non-enzymatic electrochemical glucose sensors are crucial [15].

Non-enzymatic glucose sensors are based on the concept of direct oxidation of glucose on the electrode surface. The electrochemical catalysis reaction requires the adsorption of glucose to the electrode surface by forming bonds with the unfilled d-orbitals of the catalyst. The catalytic reaction occurs on the electrode surface, where the glucose (analyte) comes into contact with the bio-receptor (recognizing the analyte). A product forms, which is recognized by the electrode surface, where the biological signal is converted into an analytical signal. 

Non-enzymatic glucose sensors may suffer from electrode blockage due to the adsorption of glucose oxidation intermediates and can be limited in the instance whereby old or faulty modified electrodes can cause activity loss, instability, and surface poisoning [16]. To enhance and promote the sensitivity of glucose sensors, direct contact of glucose with the electrode surface and fast electron transfer between the conductive electrode and electrolyte are achieved by eliminating enzymes. Non-enzymatic glucose sensors display longer-term stability than enzymatic sensors, hence the need for further research to ensure that the problems and challenges mentioned are eliminated [17,18].

## 3. Copper Oxide Nanomaterials in Glucose Detection

Copper oxide (CuO) has received particular attention because it is the simplest member of the copper compound family and is increasingly used in a number of industry sectors [19]. Copper oxide nanoparticles are inexpensive and stable in terms of their chemical and physical properties [20]. Furthermore, CuO has a high surface-to-volume ratio, which makes it highly reactive and easy to interact with other materials [21].

### 3.1. CuO Properties

Copper oxide (CuO) is a semiconductor with a band gap of 1.2–1.9 eV. CuO nanomaterials are versatile and have a wide range of applications. Their unique properties make them useful for a vast range of industries, such as healthcare, energy and electronics, and the environmental industry. Some of the key properties that make CuO attractive include:

(a) antimicrobial activity and low toxicity, making them useful for applications such as wound dressings, cancer therapy, and drug delivery [22,23,24,25],
(b) photocatalytic activity, where CuO is used to catalyze chemical reactions using photon energy in applications for water purification and environmental remediation [26,27], and
(c) high electrical conductivity for applications in electronics and sensors [28,29,30,31,32]. These properties also depend on the synthesis route employed and, hence, careful consideration of the synthesis method is critical.

### 3.2. Preparation of CuO and CuO Composites

The synthesis of CuO and CuO composites involves several methods, each with its own advantages and considerations. The most common chemically based preparation methods include chemical precipitation, the sol–gel method, and colloidal synthesis.

Chemical precipitation involves the copper salt (such as copper acetate) being dissolved in a suitable solvent, followed by the addition of a reducing agent (such as sodium hydroxide). This leads to the formation of copper hydroxide (Cu(OH)_2_) molecules, which are then thermally or chemically treated to obtain CuO nanoparticles [33,34]. Nahar et al. used chemical precipitation to produce spherical CuO nanoparticles of size 6.2 nm, which demonstrated moderate antibacterial activity [35]. 

For the sol–gel method, dissolved copper salt is added to water and/or alcohol. The mixture is then stirred and heated, leading to the formation of a gel. The gel is subsequently calcined at high temperatures to obtain CuO nanoparticles [36,37]. Sivayogam et al. presented the difference in crystallinity owing to the different calcination temperatures as the final step during the sol–gel preparation of CuO nanomaterials. Particles obtained from calcination at 700 °C were more crystalline than those obtained at 500 and 300 °C and hence presented well-defined peaks in the XRD diffractograms [38]. 

The colloidal synthesis of CuO involves the use of copper salt, a reducing agent, as well as a capping or stabilizing agent. These reactants are mixed at varying proportions depending on the requirements or specifications of the CuO nanoparticles. and the produced nanomaterials are in solution form [39,40]. Silva et al. prepared hexadecyltrimethylammonium bromide (CTAB)-stabilized CuO nanoparticles in solution. Different molar ratios of CTAB:Cu^2+^ and NaBH_4_:Cu^2+^ were explored for optimal synthesis conditions. Stable, monodispersed spherical CuO particles with hydrodynamic diameters of 36 ± 1.3 nm were obtained using molar ratios at 1:6:10 of Cu^2+^:CTAB:NaBH_4_ [41]. 

When forming CuO composites, the synthesis method typically involves the incorporation of additional materials into the CuO matrix, such as various carbon materials, precious metals, or other base metal oxides. Preparation methods include, but are not limited to, physical mixing and co-precipitation. CuO nanoparticles can be physically mixed with other materials, such as carbon nanotubes (CNTs) or graphene, by ultrasonication or mechanical mixing. This method allows for the integration of different materials; however, the material may suffer from a lack of strong interaction between the two components, the nanoparticles and the support material [42,43]. Zhao et al. prepared Cu/CNT catalysts using an ultrasonic-assisted impregnation method and they observed agglomeration of the Cu nanoparticles, which they attributed to the weak interaction between Cu species and CNTs [44]. The co-precipitation method is when the precursor materials, e.g., metal salts, are mixed with the support material before precipitation. This leads to the formation of a composite material now consisting of the metal oxide and the support [45,46]. Li et al. synthesized CuO/ZnO catalysts supported on mesoporous carbon using the co-precipitation method. The method produced evenly distributed metal oxide on the support material, and the CuO composite showed favorable catalytic activity/conversion during CO_2_ hydrogenation to methanol [47]. An example of the synthesis of CuO with metal nanoparticles is given as follows: Typically, the metal nanoparticles are synthesized by reducing the metal salt in a one-step refluxing method using various reducing agents such as ethylene glycol or citrate. The metal nanoparticle mixture then gets added to the pre-prepared CuO in DMF media and mixed. The metal–CuO nanocomposite is then obtained following centrifugation and drying in an oven under vacuum. The choice of synthesis method for CuO and CuO composites depends on factors such as desired particle size, morphology, composition, and specific application requirements. Each method offers unique advantages in terms of control over the preparation parameters and the resulting properties of the materials. Currently, there is no one optimized preparation method ideal for CuO or CuO composites for use in non-enzymatic glucose biosensors, as the technique relies on various factors for successful detection and quantification of glucose. Therefore, continuous research is needed for simple preparation methods to give optimum results for highly stable, sensitive, and selective glucose sensing. 

### 3.3. CuO and CuO Composites in Non-Enzymatic Glucose Sensors

CuO and CuO composites have been extensively studied for their application in non-enzymatic glucose sensors. These sensors are designed to detect, measure, and quantify glucose levels in biological samples such as blood, saliva, sweat, or urine without the need for enzymes, which are commonly used in enzymatic glucose sensors. CuO-based sensors offer several advantages, including high sensitivity, selectivity, stability, and cost-effectiveness as opposed to enzymatic sensors. 

#### 3.3.1. CuO/C

The high specific surface area of CuO nanomaterials allows for enhanced glucose adsorption and improved electron transfer kinetics. To further enhance the performance of the sensor, various CuO composites have been employed. These composites aim to improve factors such as sensitivity, selectivity, stability, and response time of the sensors. Some commonly used materials for CuO composites for glucose sensing include carbon nanotubes (CNTs), graphene, metal nanoparticles, and, to a lesser extent, conducting polymers. The incorporation of CNTs or graphene into CuO matrices provides several advantages. These nanomaterials possess a high surface area, excellent electrical conductivity, and good mechanical strength. They can enhance the charge transfer rate and facilitate electron transport, resulting in improved sensor performance. For example, Geetha et al. used a CNT/CuO composite for a completely enzyme-free glucose sensor. Due to its electron transport capabilities, the composite material showed excellent sensitivity and stability for glucose detection in artificial sweat. The catalytic performance of the sensor had a detection limit of 3.90 µM and a sensitivity of 15.3 mA cm^−2^ uM^−1^ [48]. Cuara et al. presented a highly sensitive and selective glucose sensor based on mole ratios of 1:0.2 weight ratios of graphene nanoplatelets to Cu_2_O and CuO composites. They reported a low glucose detection limit of 0.25 μM and a high sensitivity of 483 and 845 μA/mM cm^2^. In addition, their sensor showed a very low response to possible interferents such as uric acid, ascorbic acid, and dopamine [49]. 

#### 3.3.2. CuO/Metal Oxides

Furthermore, incorporating metal oxides, such as tin oxide (SnO_2_) or zinc oxide (ZnO), into CuO matrices can lead to improved sensor performance as well. A study conducted by Cai et al. using ZnO–CuO porous core–shell spheres in non-enzymatic glucose sensors showed a wide linear range of 0.02–4.86 mM, a sensitivity of 1217.4 μA cm^−2^ mM^−1^, and a detection limit of 1.677 μM. They attributed the overall good performance of the sensor to the individual properties of ZnO and CuO; that being, the excellent electro-oxidation ability of CuO to glucose and the good electron transfer property of ZnO, thus creating a synergistic effect [50]. More recently, Wang et al. applied their CuO–Co_3_O_4_ prickly-sphere-like composite to a non-enzymatic glucose sensor. The sensor performed relatively well; however, only had a detection limit of 21.95 μmol·L^−1^ and a sensitivity of 1503.45 μA·(mmol·L^−1^)^−1^·cm^−2^ [51]. It can be noted that although this sensor needs some improvements, it exhibited potential for practical application. 

#### 3.3.3. CuO/Metals 

Consequently, CuO composites with metal nanoparticles, such as gold (Au), silver (Ag), or platinum (Pt), have also been investigated. The electron-rich metal nanoparticles can serve as catalysts, promoting the electrochemical oxidation of glucose and enhancing the sensor’s sensitivity. A study conducted by Myung et al. involved the synthesis of a Pt-CuO nanocomposite electrode using the galvanostatic electrodeposition method. The electrode showed a positive response to glucose sensing with a sensitivity of 3812 μA mM^−1^ cm^−2^, a limit of detection of 7.5 μM, and a linear range between 0 and 0.6 mM, which was an improvement from the CuO electrode used before [52]. Viswanathan et al. used a multicore–shell Ag–CuO nanocomposite networked with CuO nanorods in a study for glucose detection. They varied the Ag:Cu atomic ratio and found that Ag–CuO (1:2.5) exhibited the best electrocatalytic activity towards glucose. The Ag–CuO (1:2.5)-modified electrode showed a sensitivity of 150.17 μA mM^−1^ cm^−2^ and a detection limit of 5 μM within a linear range of 5 μM to 30 mM [53]. The authors were satisfied with the detection limit of 5 μM, as it is much lower than the physiological concentration of glucose. Chakraborty et al. decorated hydrothermally grown CuO nanorods (NRs) with gold nanoparticles (Au) and deposited these Au–CuO NRs onto a fluorine-doped tin oxide (FTO) glass substrate. The electrode showed a positive response to glucose detection. Compared to the pristine CuO NRs electrode, the Au–CuO NRs electrode showed improved sensitivity of 2009 μA cm^−2^ mM^−1^ from 1331 μA cm^−2^ mM^−1^, within a linear range of 5 μM to 1.325 mM. The sensitivity had increased 1.5-fold upon incorporation of the Au nanoparticles. Moreover, the limit of detection also improved from 0.25 μM with the CuO NRs electrode to that of 0.17 μM with the Au–CuO NRs electrode [54]. Additionally, the metal nanoparticles can act as nano-sized electrodes, providing a large surface area for more glucose adsorption and contributing to rapid readings that are more precise. The authors of the Au–CuO NRs electrode reported that it was 1.5 times faster towards glucose detection, stating that response time with prestine CuO NRs was 2.5 s and that of Au–CuO_2_ NRs was 1.6 s [54]. Table 1 gives examples of recently reported noble metal-CuO composite electrodes used for glucose sensing in non-enzymatic sensors. The reported values give a clear indication that these types of composites have the true potential to be applied in glucose monitoring for the maintenance of diabetes.

#### 3.3.4. CuO/Polymeric Nanocomposites

Conducting polymers, such as polyaniline (PANI), have also been utilized in CuO composite-based sensors. The polymers not only provide a supporting matrix for CuO but also contribute to the overall sensor performance through their electrochemical properties. The combination of CuO with conducting polymers can enhance the electron transfer rate, increase the sensor’s stability, and improve its selectivity towards glucose. Using a CuO-PANI nanofiber-modified fluorine-doped tin oxide (FTO) electrode for glucose sensing, Esmaeeli et al. reported a detection limit of 0.24 μM within a linear range of 0.28 μM to 4.6 mM and a sensitivity of 1359 μA mM^−1^ cm^−2^ [63]. Ghanbari and Babaei explored a ternary NiO/CuO/polyaniline composite for the detection of glucose. The sensor demonstrated a good linear relationship in the range of 20–2500 μM (correlation coefficient = 0.9979), and the detection limit of glucose at the electrode was 2.0 μM [64]. These results demonstrate that the composite material has the potential to be applied in non-enzymatic glucose detection systems. Bringing the above-mentioned properties brought by various elements within a sensor, Fang et al. constructed a 3D porous structured polyaniline/reduced graphene oxide/copper oxide decorated system on a platinum electrode (Pt/PANI/rGO/CuO) in the hope of achieving maximum sensitivity, linearity, selectivity, repeatability and stability of the sensor. The fabricated electrode illustrated higher electrocatalytic activity than glucose, exhibiting a high sensitivity of 1252 μA mM^−1^ cm^−2^, a fast response time of less than 3 s, a detection limit of 1.5 μM, and a linear range from 0 mM to 13 mM, thus showing potential to be used for glucose detection [65]. 

## 4. Copper Sulfide Nanomaterials in Glucose Detection

**Figure 3:**
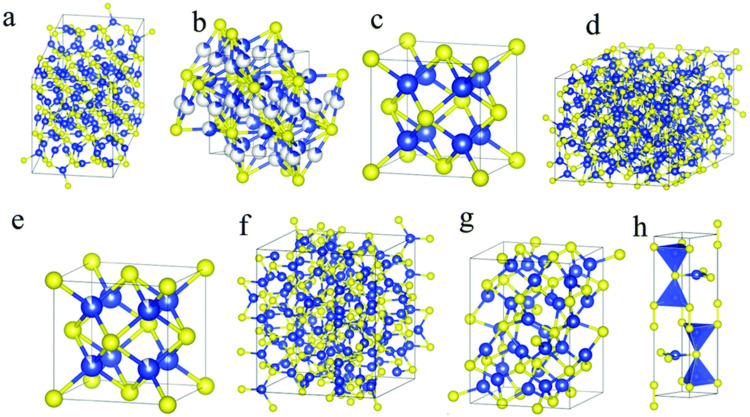
Crystal structures of the different phases of copper sulfide where (**a**) low chalcocite, (**b**) high chalcocite, (**c**) cubic chalcocite, (**d**) djurleite, (**e**) digenite, (**f**) roxbyite, (**g**) anilite, and (**h**) covellite phases. Blue and yellow spheres represent copper and sulfur atoms, respectively [74].

Due to their excellent electrical conductivity, abundance, ability to promote electron transfer reactions with biomolecules, and low cost compared to other materials, copper sulfides have been steadily investigated [9,66,67]. The choice of Cu_x_S_y_-based sensors is due to their exceptional sensitivity, long-term stability, and short response time [68]. Copper sulfide nanoparticles can be synthesized to form a variety of stoichiometric phases, from copper-rich to copper-deficient phases, which depend on the reaction conditions used [69]. The crystal structure of the various phases depends on the packing of the sulfur in the lattice (Figure 3), and the known phases are: CuS (covellite), Cu_1.96_S (djurleite), Cu_1.8_S (digenite), and Cu_2_S (chalcocite), and have potential applications such as batteries, capacitors, sensors, and photothermal conversion [9,70,71]. The different stoichiometric phases can be produced through simple chemical and physical methods such as chemical vapor deposition, solvothermal, co-precipitation, microwave, and hydrothermal synthetic routes [72,73].

### 4.1. Cu_x_S_y_ Properties

Copper sulfides, identified as p-type semiconductor materials due to the copper vacancies within the lattice, have been studied widely due to their wide range of applications, from energy to biomedical fields, as well as their non-toxic nature [75]. The short Cu–Cu distance, such as metallic Cu–Cu bonding as well as the short Cu–S distance resulting in close packing, accounts for their high electrical conductivity, specifically in Cu_2_S and Cu_1.94_S [76]. Depending on the stoichiometry of the copper sulfide, the optical band gap varies from 1.2 to 2.35 eV [77,78,79]. Covellite (CuS) copper sulfides exhibit good electrical conductivity of 10^−3^ S cm^−1^ attributable to their metal-like electrical conductivity [80,81]. Additionally, chalcocites (Cu_2_S), one of the polymorphs of CuS, also possess interesting metal-like electrical conductivity. These copper sulfides have therefore been explored as potential candidates for electrochemical glucose sensors [82]. Combining copper sulfides with other materials to form hybrid nanostructures allows for the manipulation of these properties and has shown enhanced activity in several applications.

### 4.2. Preparation of Cu_x_S_y_ and Cu_x_S_y_ Composites

Nanodimensional copper sulfides can be prepared through several methods, producing different compositions and phases. The synthesis of well-controlled Cu_x_S_y_ monodispersed nanoparticles with defined morphology and sizes remains a challenge. The hydrothermal process allows for the investigation of these nanoparticles by varying parameters such as the precursors used, the temperature of the reaction, reagent ratios, pH, reaction time, and so on [5,67,83]. This process does not require high energy, temperature, vacuum, pressure, or cooling systems and enables an increase in reactant solubility. Hydrothermal and solvothermal methods are among the most commonly utilized methods for synthesizing nanomaterials. The Cu_7_S_4_–CuS mixture, CuS, and Cu_9_S_5_ were synthesized via the solvothermal method by varying the S and Cu precursor ratios [84]. The drawbacks of this method are the use of expensive autoclaves, the inability to observe the crystals as they grow, and the fact that it is not entirely reliable and reproducible [85]. Another method commonly used for synthesizing monodispersed, high-quality copper sulfides from surfactants and a mixture of organic solvents is the one-pot hot injection method. The method involves the sudden addition of “cold” reactants (room temperature) into the hot solvent, forming a sudden burst of nucleation and the growth of nuclei under optimum reaction conditions [86,87]. One of the fastest strategies for decomposing Cu–S complex precursors and synthesizing copper sulfide nanocrystals is the use of microwave irradiation by thermolysis in a frequency range of 0.3 to 2.45 GHz. This microwave frequency range allows the conversion of electromagnetic energy to thermal energy, including the chemical reaction resulting in the synthesis of nanostructured materials [88]. Microwave irradiation has disadvantages such as the use of expensive equipment, being unsuitable for scaling up, and not being feasible for reaction monitoring [89]. A simple, low-cost, and high-throughput technique to fabricate copper sulfides directly on the substrate is electrochemical deposition or anodization. The nanostructures are also grown on Cu–foil or Cu–substrates acting as the anode and Ti-metal as the cathode in the voltage range of 1.5–8 V in Na_2_S aqueous solution [90]. This method is costly and displays other limitations, such as non-conformal growth on non-planar surfaces. Other restrictions include allowed morphologies and nanomaterial dimensions [91,92].

Microemulsion is a method used to prepare uniform and size-controlled metal particles. The method is an isotropic dispersion of two immiscible liquids, such as water and oil, with surfactant molecules stabilizing the liquids at the water/oil interface [93,94]. The system is dependent on the nature of the dispersed liquid, where the dispersion liquid is classified as either oil in water (O/W)–oil droplets exist dispersed in bulk water and vice versa for water in oil (W/O). The main drawback of the microemulsion method is the narrow linear range around micromolar concentrations, which requires predilution of the sample [66]. The other drawback is the use of large amounts of surfactants [95]. Bulk production of materials can be synthesized without using high temperatures, high pressures, or prolonged reaction conditions via a sonochemical method [96]. The method is also used in the modification of polymers/biopolymers. One of the disadvantages of this method is its low efficiency [95].

Cu_x_S_y_ nanomaterials have been reported to be important in numerous bio-sensing applications. Therefore, techniques for both chemical and physical characterization of these synthesized nanomaterials are crucial. Characterization can be performed using several techniques that provide optical, elemental, and structural properties, namely X-ray diffraction (XRD), scanning electron microscopy (SEM), energy dispersive X-ray spectroscopy (EDS), UV–visible and photoluminescence (PL) spectroscopy, transmission electron microscopy (TEM), atomic force microscopy, Fourier transform infrared spectroscopy (FTIR), and dynamic light scattering (DLS). 

### 4.3. Cu_x_S_y_ and Cu_x_S_y_ Composites in Glucose Sensors

In addition to the stoichiometry of the copper sulfide nanomaterial, the performance of glucose electrooxidation also depends on the shape, composition, and active sites of the material. Zhang et al. reported the use of CuS nanotubes (CuS NTs) successfully prepared in an O/W microemulsion system under low temperature as a non-enzymatic glucose sensor [66]. The CuS nanotubes displayed electrocatalytic activity towards glucose oxidation with a sensitivity of 7.842 µA mM^−1^ and a linear range in the glucose concentration up to 5 mM. The as-prepared CuS nanotubes were made up of CuS nanoparticles and provided a large surface area, active points, and electron transfer passage, which led to ease of communication with the surface of the electrode. Similarly, CuS NTs made up of CuS nanoparticles were successfully prepared by Qian et al. in situ on a Cu electrode by a simple self-sacrificial template method and investigated for glucose electrooxidation [97]. The CuS NTs were grown on Cu electrodes in situ to avoid sonication, which may destroy the structure of the NTs, affecting their electrocatalytic activity. The glucose sensor exhibited a detection limit of 45 nM and two wide linear ranges (0.2 µM to 2.5 mM and 2.5 mM to 6 mM) with sensitivities of 3134 µA mM^−1^ cm^−2^ and 2205 µA mM^−1^ cm^−2^, respectively. This phenomenon was observed to be the rapid diffusion of glucose into the CuS NTs at lower glucose concentrations, hence the current rapidly increasing with the glucose concentration. Whereas the adsorption of the intermediate hindered glucose diffusing into the CuS NTs by decreasing the active sites, hence the decrease in sensitivity at a higher glucose concentration.

Well-designed nanostructures of copper sulfides can increase the surface active sites and therefore enhance the electrocatalytic activity. This can be achieved by preparing hybrid materials to obtain a combination of the properties of the materials involved, resulting from the synergistic component effects [98].

A large contact area and a porous and hollow inner surface are favorable for allowing contact between the modified material/electrode and glucose. This was observed by Lin and co-workers, where sphere-like copper sulfide (CuS) microcrystals were constructed by nanosheets aligned vertically on the spherical surface, resulting in a hollow inner and porous surface [99]. The sensitivity of the sphere-like CuS microcrystal-modified electrode towards the non-enzymatic oxidation of glucose was 117.3 µA mM^−1^ cm^−2^ with a linear range of 0.1–12,000 µM and a limit of detection of 0.19 µM. The CuS microflower (MF) superstructure-based non-enzymatic sensor developed by Radhakrishnan et al. using a simple and facile method without surfactants or templates successfully oxidized glucose [100]. The CuS MF sensor showed a sensitivity of 1007 µA mM^−1^ cm^−2^, a detection limit of 2.0 µM, and a glucose concentration range of 0.02–5.4 mM. The electrocatalytic activity was due to the unique structure and high surface areas, which reduced the diffusion length of glucose, improving the electron transfer passage between glucose and the electrode.

#### 4.3.1. CuS/C Nanohybrids

Graphene has been widely used in the construction of sensors due to its high electrical conductivity, good catalytic activity, and large specific area. However, graphene has been reported to form disorderly stacked structures that reduce the specific surface area, subsequently reducing its catalytic activity. Therefore, the formation of composites with other nanomaterials can fully optimize their surface area. Karikalan and co-workers prepared copper sulfide and sulphur-doped reduced graphene oxide nanocomposite (S-rGO/CuS) via a sonochemical method [101]. Sulphur-reduced graphene was utilized for its physicochemical properties and CuS for its electrocatalytic activity, making the composite an efficient catalyst for glucose oxidation. The structure of the as-prepared S-rGO/CuS changed from covellite to digenite phase, presenting a detection limit of 32 nM, sensitivity of 429.4 µA mM^−1^ cm^−2^ and a wide linear range of 0.0001–3.88 and 3.88–20.17 mM. Later, Yan and co-workers successfully synthesized copper sulfide nanoflake-reduced graphene oxide (rGO/CuSNFs) nanocomposite, where the presence of CuS nanocrystals reduced the restacking of graphene, allowing full utilization of the active surface sites [102]. The nanocomposite was prepared via a one-pot hydrothermal treatment where in situ generation of CuS nanoflakes and reduction of GO occurred simultaneously. The as-prepared nanocomposite exhibited high electrocatalytic activity towards the oxidation of glucose, with a fast response time, a detection limit of 0.19 µM, a wide linear range from 1 to 2000 µM, and a sensitivity of 53.5 µA mM^−1^ cm^−2^. The excellent catalytic activity of CuS nanocrystals and the excellent conductivity of rGO created a synergetic effect that enhanced the sensitivity of the nanocomposite. Chemical stability and catalytic activity were further supported by the rGO nanosheet, inhibiting agglomeration of the supported nanostructures and providing a conductive channel.

Hollow nanostructured nanomaterials have been reported to have enhanced electrocatalytic properties due to the void space inside the distinct shell having a high specific surface area, shell permeability, and volume buffer. This is reported by Cao et al., where they observed that the amperometric current response for glucose oxidation increased in the order Cu_2_O nanospheres < Cu_2_S < CuS < Cu_4_S_7_ hollow nanospheres [103]. The Cu_4_S_7_ hollow nanospheres exhibited the highest sensitivity of 3728.7 µA µM^−1^ cm^−2^ compared to the other Cu_x_S hollow nanospheres and a wide linear concentration range from 1.0 µM to 2.0 mM with a limit of detection of 0.023 µM in alkaline medium. The high electrocatalytic activity of Cu_4_S_7_ hollow nanostructures was influenced by their large specific surface area, good electron conductivity, and the presence of an inside-out or void-like open structure configuration, which allows access to large amounts of glucose molecules to the inner and outer surfaces.

Research on naturally derived carbon materials, which are renewable, cost-effective, and eco-friendly, has received attention over the years. These natural biomolecules have been utilized in electrochemical applications due to their excellent conductivity, electrical stability, and large surface area [104]. Xanthan gum (XG) is a polysaccharide produced by bacteria that presents unique chemical and physical properties and has strong binding properties with water and organic/inorganic materials [105]. Keerthi et al. reported urchin-like CuS grown on XG-derived carbon nanofibers, resulting in a biocompatible CuS/XGCNFs hybrid material for the non-enzymatic glucose sensing of glucose [106]. The hybrid material was studied for electrocatalytic glucose oxidation and achieved a sensitivity of 23.7 µA mM^−1^ cm^−2^ and a limit of detection of 0.019 µM. The hybrid material’s sensitivity is attributed to its unique architecture, which maximizes electron transportation. 

#### 4.3.2. Metal and Non-Metal Doped CuS Nanomaterials

Copper sulfide nanosheets can be used to effectively stabilize nanoparticles through bond formation with S at the surface, improve charge transfer ability, and are an active substrate for anchoring other active catalysts on their surface. Mai et al. reported 2D Cu_x_S nanosheets synthesized on a 3D copper foam (3DCF) and then prepared a sensor based on the electrodeposition of gold nanoparticles (Au NPs) on the Cu_x_S nanosheets, forming Au–Cu_x_S/3DCF [107]. The sensor showed high activity towards glucose oxidation with a sensitivity of 0.059 mA µM^−1^ cm^−2^, a detection limit of 7.62 µM, and a wide linear detection range of 1.98–976.56 µM. Au nanoparticles are known for their excellent catalytic properties for increasing current responses in several electrochemical reactions. However, the catalytic performance can be reduced due to inadequate interaction between the substrate and Au, leading to the reaggregation and dissolution of Au NPs during operation. Therefore, incorporating abundant Au NPs onto the large surface area of Cu_x_S nanosheets produced a synergistic effect, enhancing the electroactive sites, adjusting the adsorption energy, improving electrolyte penetration/ion diffusion, and excellent charge transfer, resulting in high activity. 

Dendritic structures and combinations are used to increase the conductivity and catalytic activity, especially in non-enzymatic glucose sensors; hence, researchers have designed and prepared Cu_x_S dendrites and their nanocomposites with high-conducting nanomaterials. Xu and co-workers prepared a dendritic Cu–Cu_2_S nanocomposite by an in-situ electrodeposition method onto a GCE without binders or any post-treatment for a non-enzymatic glucose sensor [108]. The electrocatalytic activity of the Cu–Cu_2_S/GCE sensor was investigated towards glucose oxidation and exhibited a sensitivity of 5.02 mA mM^−1^ cm^−2^ with a limit of detection of 0.33 µM in the concentration range from 0.1 µM to 0.5 mM. The catalytic activity was attributed to the large surface area and good electron transfer passage exhibited by the dendritic nanostructure. Kim et al. demonstrated the effect of a large active surface area on the electrochemical performance of their non-enzymatic glucose sensor. The group prepared CuS dendrite by electrodeposition and vapor-phase sulfurization and investigated its electrocatalytic activity towards glucose oxidation [9]. The CuS dendrite presented a sensitivity of 8337 µA mM^−1^ cm^−2^ in a wide linear range of 0.001–4.9 mM with a detection limit of 0.05 µM. The performance was due to the high ability of the dendritic structure to transport electrons and its large surface area.

Recently, Sharma and co-workers successfully synthesized a nitrogen (N) and sulphur (S) co-doped chitosan polymer matrix-derived composite (CuS/NSC) via a simple one-step hydrothermal technique using a copper complex of chitosan polymer of low cost [109]. The electrocatalytic activity of the CuS/NSC sensor was investigated for glucose oxidation. The sensor exhibited a linear range of 160 µM to 11.76 mM, a sensitivity of 13.62 mA mM^−1^ cm^−2^ and a low detection limit of 2.72 µM with excellent linear response. N- and S-doped carbon spheres (NSC) were utilized as a supporting matrix to anchor CuS nanoparticles and enhance the electrochemical performance of the glucose sensor. The heteroatom-doped carbon sphere material helped increase the electrocatalytic activity by enhancing the wettability of the electrode material, electronic conductivity towards the electrolyte, and storage capacity. The material also provided aid to the redox reaction occurring on the surface of the electrode, which may be due to the lone pair of electrons present in the N and S atoms.

#### 4.3.3. CuS-Based Mixed Metal Oxides

Copper oxides (CuO or Cu_2_O) have been widely investigated for glucose sensing, as discussed earlier. Wei et al. prepared CuS/Cu_2_O/CuO electrodes by modifying Cu_2_O/CuO nanowire arrays (NWAs) with CuS nanosheets and fabricated them on Cu foil by in-situ growth and successive ionic layer adsorption and reaction (SILAR) methods without using any binders [110]. The in-situ growth was reported to ensure a good connection between copper oxides and copper substrate, decreasing the inner resistance and promoting electron transfer between the active material and highly conductive substrate [111]. The NWAs were utilized as they possess large active surface area. The modified electrode was employed for non-enzymatic glucose sensing and presented a sensitivity of 4262 µA mM^−1^ cm^−2^ in the range from 0.002–4.096 mM. The CuS nanosheets enhanced the electrooxidation by increasing the active area of the Cu_2_O/CuO/Cu electrode towards glucose; hence, the amperometric response of the optimized CuS/Cu_2_O/CuO/Cu electrode was twice that of the Cu_2_O/CuO/Cu electrode. Mallick and co-workers reported on a copper sulfide (Cu_2_S)-based non-enzymatic glucose sensor with a detection limit of 2.42 μM, much lower than the normal glucose level in the physiological system, and presented a sensitivity of 38.21 µA mM^−1^ cm^−2^ [112]. The hexagonal copper sulfide nanoparticles were stabilized by polyaminobenzoic acid by applying a single pot “in situ polymerization and composite formation” protocol and were almost evenly distributed within the polymer matrix. 

Copper-rich and copper-deficient sulfides have shown potential in the electrochemical oxidation of glucose due to their excellent properties. Taking advantage of this, Huang and co-workers reported a non-enzymatic glucose sensor based on a hollow-structured copper sulfide/cuprous sulfide (CuS/Cu_2_S) hybrid prepared using a one-pot solvothermal method [113]. The results showed that the integrated electrode displayed excellent electrocatalytic performance towards the oxidation of glucose with a high sensitivity of 321.4 µA mM^−1^ cm^−2^, a low detection limit of 1.1 µM, and a wide linear range of 3.0–1100 µM. The high electrocatalytic activity of the hybrid was attributed to the synergy between CuS and Cu_2_S through obtaining active sites with a large surface area, porous exteriors, and a hollow interior structure of Cu_2_S; and lastly, the hollow-structured hybrid provided diffusion channels that facilitated the mass transport oxidation of glucose.

The reported non-enzymatic glucose sensors discussed above (see Table 2) have shown good sensitivity, selectivity, anti-interference, and accuracy in real sample analyses and therefore showed potential for accurately monitoring glucose in biological samples at low cost.

## 5. Comparison of Different Substrates for Use as Electrodes

Substrate selection plays a crucial role in the performance of screen-printed electrodes (SPEs) used in non-enzymatic glucose sensors. Different substrates can exhibit variations in conductivity, surface properties, mechanical strength, and compatibility with manufacturing processes. Commonly used substrate materials for SPEs in non-enzymatic glucose sensors include ceramic substrates, glass substrates, polymer substrates, and paper substrates. Each class of these substrates offers its own advantages and disadvantages. Ceramic substrates (alumina and alumina-based) have high mechanical strength and durability, good thermal and chemical stability, excellent electrical insulation properties, and are compatible with thick-film printing techniques. On the contrary, ceramic substrates are relatively more expensive compared to other substrate materials, and they have limited flexibility. Glass substrates have excellent thermal stability and chemical resistance, have shown good mechanical strength, have a flat and smooth surface that allows for precise printing and electrode fabrication, and are suitable for high-temperature processing. However, some of their disadvantages include their brittle nature, making them more prone to breakage, higher cost compared, limited flexibility, and lowered conductivity than ceramic substrates. Polymer substrates (made of either polyimide or polyester, etc.) have key and attractive advantages such as low cost and wide availability, good flexibility and bendability, enabling the production of flexible or wearable sensors, being lightweight and portable, and compatibility with both thick-film and thin-film printing techniques. On the other hand, some of the disadvantages include limited thermal stability, lower mechanical strength compared, and the potential for leaching of polymer additives or plasticizers, which may interfere with the sensor performance. The fourth type of substrate is paper-based substrates, which have advantages similar to those of polymer-based substrates, but moreover, they are flexible and lightweight, suitable for disposable or portable applications, and most importantly, they are environmentally friendly and have low-cost fabrication printing techniques. Despite these favorable traits, paper-based substrates have some shortfalls, which include limited thermal and chemical stability, surface roughness and porosity, which can affect electrode quality and performance, lower mechanical strength, and higher risk of moisture absorption, which may affect the electrode’s stability and performance. Table 3 gives some examples of various substrates for potential use in electrochemical glucose sensing. It is important to note that the performance of the electrodes differs in terms of sensitivity, linear range, and the limit of detection. Therefore, the selection of a substrate for SPEs depends on various factors, including the specific requirements of the non-enzymatic glucose sensor, the desired performance characteristics, the manufacturing processes, and the intended application. Each substrate material has its own advantages and disadvantages, and the choice should be made based on the specific needs of the sensor design and target application.

## 6. Limitations of Copper Oxides and Sulfides in Glucose Detection

While CuO and CuS have shown promise in glucose detection, they also have certain limitations that need to be considered. One of the main challenges in non-enzymatic glucose detection using copper-based materials is the interference from other analytes present in complex biological samples. The selectivity of copper-based materials towards glucose can be compromised in the presence of interfering species such as ascorbic acid, uric acid, and dopamine. These species can undergo similar electrochemical reactions on the surface of copper-based materials, leading to false-positive signals or reduced accuracy. Copper-based materials, including CuO and CuS, may have a limited detection range compared to enzymatic glucose sensors. The linear range for glucose detection using copper-based materials can be relatively narrow, limiting their ability to accurately detect a wide range of glucose concentrations. This limitation can be problematic for applications that require high sensitivity and a broad dynamic range. Degradation or instability over time is also a challenge for these copper-based electrode systems, and this leads to a decrease in their electrochemical performance. Furthermore, the main component of these electrode systems is copper, which is a heavy metal. Possible leaching of these nanomaterials into the environment upon disposal needs to be carefully considered and taken into account. Therefore, the disposal and management of waste containing these copper-based materials should be handled with due diligence to prevent environmental contamination.

## 7. Conclusions and Future Outlook

Nanoscience and nanotechnology have contributed to the various developments of sophisticated health-related products over the last decade. As the number of diabetic cases continues to increase rapidly, there is an urgent need to design and develop highly advanced glucose devices. Nanomaterials that are used in health applications have been evaluated for their structural, morphological, and catalytic properties. Hence, CuO and CuS nanomaterials have been examined for the development of non-enzymatic glucose sensors and hold several promising possibilities. The glucose oxidation occurs at the electrode/electrolyte interface, which is governed by direct electron transfer kinetics. The use of CuO and CuS as individual electrode materials has resulted in sluggish electron and charge transfer kinetics, which has led to the incorporation of other materials (carbon, metal, metal oxides, non-metal dopants, and polymers) to ensure rapid analytic response and high sensitivity. The nanocomposites have contributed differently, with some only improving electron transfer while the analytical signal remains very low and vice versa. This shows that further functionalization and continuous optimization are still required. For instance, incorporating specific functional groups to modify the surface of the nanomaterials can help mitigate interference from other species commonly found in biological samples. This would enable more accurate and specific glucose measurements. Additionally, there are few studies on modeling of CuO and CuS-based nanomaterials on electrode surfaces in order to ascertain their binding to glucose molecules. Understanding the mechanism will determine the choice of support material to further enhance the electrocatalytic behavior of the electrode material. Therefore, available catalytic sites and various supports that exhibit better surface area for uniform dispersion should be prioritized to achieve enhanced glucose sensitivity. These nanocomposites should be prepared using synthetic methods that are scalable with minimum upscaling effects and improved stability. The particle size should also be optimized for consistency. Such efforts will mostly make the process of diagnosis easier, quicker, and less invasive.

Additionally, structural engineering that can result in a Cu-based nanocomposite that consists of unfilled d-orbitals and unpaired d-electrons should be explored. This can give rise to the formation of stable multi-oxidation systems that can undergo various redox reactions, thus leading to direct electrocatalytic activities with minimal intermediate generation.

More importantly, significant research has to be undertaken in order to obtain CuO and CuS ink formulations for use in the development of printed electrodes on various substrates, which will be achieved by understanding the chemical interaction between the substrate and the Cu-based conductive inks and optimizing them in such a way that the possibility of metal leaching will be reduced.

Furthermore, intense studies have been conducted on the evaluation of the performance of the electrode as wearable sensors that utilize saliva, sweat, etc. The sensor device can be designed in such a way that it contains a pre-concentration step, which can then provide high glucose readings. Additionally, smart engineering efforts for the integration of bioelectronic pH control into glucose sensors should be prioritized in order to further enhance the operation of the glucose sensor in biological fluids.

## Figures and Tables

**Table 1:** Additional recent reports showing noble metal-CuO composites in non-enzymatic electrochemical glucose sensing.

Electrode Material	Sensitivity (µA mM^−1^ cm^−2^)	Linear Range (mM)	Detection Limit (µM)	Reference
Ag–CuO/rGO	214.37	10–28	0.76	[55]
Ag–CuO	2528.6	0.01–1	1.5	[56]
Ag@CuO	3763.44	1–9.2	0.006	[57]
Ag/CuO/MLG	1527	0.01–6.0	3.8	[58]
Pt-CuO/GPE	2035	3.125–18.75	0.1	[59]
Au/CuO NWs	4398.8	0.0005–5.9	0.5	[60]
Au@Cu_2_O	1601	0.005–2.1	0.6	[61]
Au/CuO	63.66	3–18	0.22	[62]
Au/CuO	172.45	0.002–1	0.22	[62]

rGO: reduced graphene, MLG: multilayer graphene, GPE: graphite pencil electrode, NWs: nanowires.

**Table 2:** Reported Cu_x_S_y_-based non-enzymatic glucose biosensors.

Electrode Composition	Sensitivity	Linear Detection Range (mM)	Limit of Detection (µM)	Reference
CuS nanotubes	7.842 µA mM^−1^	0.00005–0.005	-	[66]
CuS dendrite	8337 µA mM^−1^ cm^−2^	0.001–4.9	0.05	[9]
CuS nanotubes/Cu	3135 µA mM^−1^ cm^−2^	0.0002–2.5	0.045	[97]
Sphere-like CuS microcrystals	117.3 µA mM^−1^ cm^−2^	0.0001–12	0.015	[99]
CuS MF	1007 µA mM^−1^ cm^−2^	0.02–5.4	2.0	[100]
Cu_7_S_4_	3728.7 µA mM^−1^ cm^−2^	0.001–2.0	0.023	[103]
S-rGO/CuS	429.4 µA µM^−1^ cm^−2^	0.0001–20.17	0.032	[101]
rGO/CuSFs	53.5 µA µM^−1^ cm^−2^	0.001–2	0.19	[102]
CuS/Cu_2_O/CuO/Cu	4262 µA µM^−1^ cm^−2^	0.002–4.096	-	[110]
CuS/Cu_2_S	321.3 µA µM^−1^ cm^−2^	0.003–1.1	1.1	[113]
CuS/XGCNFs	23.69 µA µM^−1^ cm^−2^	0.158–1.221	0.019	[106]
Cu_2_S	38.21 µA µM^−1^ cm^−2^	-	2.42	[112]
Au–Cu_x_S/3DCF	0.059 mA µM^−1^ cm^−2^	0.00198–0.97656	7.62	[107]
Dendritic Cu-Cu_2_S	5.02 mA mM^−1^ cm^−2^	0.0001–0.5	0.33	[108]
CuS/NSC	13.62 mA mM^−1^ cm^−2^	0.16–11.0	2.72	[109]

**Table 3:** Table showing various substrates used in non-enzymatic glucose sensing applications.

Substrate	Electrode Material	Sensitivity (μA mM^−1^ cm^−2^)	Linear Range (µM)	Detection Limit (µM)	Reference
Glass	Cu on glass	719	10–1000	1.97	[114]
Glass	Cu on glass	145.521	10–200	2.87	[115]
Glass-ceramics	Cu on glass-ceramics	911	3–1000	0.75	[114]
Glass-ceramics	Cu on glass-ceramics	1110	3–3000	0.91	[116]
Polyimide (PI) foil	Graphene-Cu on PI	1518	1–4540	0.35	[117]
Polyimide film	Glucose oxidase/chitosan-modified graphene	43.15	0–8000	431	[118]

## Data Availability

Data available on request due to restrictions e.g., privacy or ethical.
